# Trends in the Use of Weightbearing Computed Tomography

**DOI:** 10.3390/jcm13185519

**Published:** 2024-09-18

**Authors:** Alessio Bernasconi, Yanis Dechir, Antonio Izzo, Martina D’Agostino, Paolo Magliulo, Francesco Smeraglia, Cesar de Cesar Netto, International Weightbearing CT Society, François Lintz

**Affiliations:** 1 Trauma and Orthopaedics Unit, Department of Public Health, University of Naples Federico II, 80131 Naples, Italy; izzoantonio1992@gmail.com (A.I.); martidag93@gmail.com (M.D.); pao.magliulo@studenti.unina.it (P.M.); francesco.smeraglia@gmail.com (F.S.); 2 Department of Orthopaedic and Trauma Surgery, Centre Hospitalier Universitaire (CHU) de Toulouse, 31300 Toulouse, France; yanisdechir@gmail.com; 3 Department of Orthopedic Surgery, Duke University, Durham, NC 27708, USA; cesar.netto@duke.edu; 4 Department of Foot and Ankle Surgery, Ramsay Healthcare, Clinique de l’Union, 31240 Saint Jean, France; dr.f.lintz@gmail.com

**Keywords:** weightbearing, computed tomography, cone beam, three-dimensional, imaging

## Abstract

**Background:** This review aimed to critically appraise the most recent orthopedic literature around cone beam weightbearing computed tomography (WBCT), summarizing what evidence has been provided so far and identifying the main research trends in the area. **Methods:** This scoping review was performed on studies published between January 2013 and December 2023 on the Pubmed database. All studies (both clinical and nonclinical) in which WBCT had been used were critically analyzed to extract the aim (or aims) of the study, and the main findings related to the role of this imaging modality in the diagnostic pathway. **Results:** Out of 1759 studies, 129 were selected. One hundred five manuscripts (81%) dealt with elective orthopedic conditions. The majority of the analyses (88 studies; 84%) were performed on foot and ankle conditions, while 13 (12%) studies looked at knee pathologies. There was a progressive increase in the number of studies published over the years. Progressive Collapsing Foot Deformity (22 studies; 25%) and Hallux Valgus (19 studies; 21%) were frequent subjects. Twenty-four (19%) manuscripts dealt with traumatic conditions. A particular interest in syndesmotic injuries was documented (12 studies; 60%). **Conclusions:** In this review, we documented an increasing interest in clinical applications of weightbearing CT in the orthopedic field between 2013 and 2023. The majority of the analyses focused on conditions related to the foot and the ankle; however, we found several works investigating the value of WBCT on other joints (in particular, the knee).

## 1. Introduction

During the last decade, an increasing number of studies have investigated the role of cone beam weightbearing computed tomography in the management of orthopedic conditions [1,2]. The advantages of this recent technology essentially related to the ability to obtain a tri-planar and three-dimensional image acquisition of musculoskeletal structures during physiological stances and with a reduced amount of radiation (cone beam technology instead of traditional fan beam tomography [3,4,5,6]) have been widely discussed in the literature and have led researchers from all over the world to investigate how this would translate in terms of clinical benefit for the patients [1,7,8,9]. The first machines (after those introduced in the maxillofacial area [10,10,11,12,13,14,15,16,17,18,19,20,21,22,23,24,25,26]) were developed to scan the foot and ankle, as regions with high anatomical complexity (28 bones and related joints) where the biases associated with bi-dimensional imaging (especially overlapping contour lines due to superimposition of bony structures and risk of inaccurate measurements related to rotation bias of the radiographic source) have been particularly felt by the orthopedic community [1]. The technological advancements have enabled industries to provide more recent machines to scan the whole lower limb including hips and knees and parts of the upper limb as well (through rotation of the gantry). After the first review on the topic published by Barg et al. in 2017 [27], some other reviews have documented the general interest in this field, highlighting the value of this imaging technology in different orthopedic conditions, its limitations and potential future perspectives [28,29,30,31,32].

With this background, we performed a scoping review of the literature aiming to identify trends in research on the role of weightbearing computed tomography in the management of orthopedic conditions. We hypothesized that (1) an increased use of WBCT for complex three-dimensional deformities could be observed and that (2) new applications to enhance the status of knowledge of musculoskeletal diseases could be identified.

## 2. Materials and Methods

### 2.1. Protocol and Registration

This scoping review was designed according to the PRISMA-ScR checklist (Preferred Reporting Items for Systematic Reviews and Meta-analyses Extension for Scoping Reviews) and followed the 6-stage methodological frameworks of Arksey and O’Malley, as found in the previous orthopedic literature [33]. The study protocol was registered on the Open Science Framework database (https://doi.org/10.17605/OSF.IO/76F59).

### 2.2. Eligibility Criteria

The inclusion criteria for this review were as follows: studies reporting data on validated or potential applications of cone beam weightbearing CT machines, both as clinical or in vitro (cadaveric or biomechanical) studies, published from 2013 to 2023; prospective and retrospective cohort studies and technical notes; English-language articles; full-text availability, either online or after direct contact with the authors. Case reports, letters to the editor, instructional courses, expert opinions and studies on animals were excluded. References from previous narrative or systematic reviews were analyzed and extracted if indicated.

### 2.3. Information Sources and Search

A systematic search was conducted on PubMed from 2013 to 31 December 2023, with the following Boolean operator: ((weightbearing) AND (ct)). Two reviewers (A.B. and A.I.) independently screened the results of the research; then, the full texts of eligible studies were analyzed. Disputes were resolved by the senior author (F.L.).

### 2.4. Data Charting and Items

Data were charted independently by 2 investigators (A.B. and A.I.), and results were compared between the investigators to verify that no data had been missed. Data extracted were the year of publication, the type of study, the level of evidence provided, the sample size of the study group, the body segment investigated (foot, ankle, knee, hip or other joints) and the medical condition evaluated using WBCT.

### 2.5. Critical Appraisal of Included Studies

Quality assessment of both comparative and non-comparative studies was performed using the Methodological Index for Non-Randomized Studies (MINORS) criteria. This checklist covered the following eight categories to assess non-randomized controlled trials (NRCTs): clearly stated objectives, the inclusion of consecutive subjects, prospective collection of data, appropriate endpoints, unbiased assessment of the study endpoints, a follow-up period in line with study objectives, loss to follow-up less than 5% and a prospective sample size calculation [34]. Each of these questions can be answered with “not indicated” (0 points), “indicated but insufficient” (1 point), or “indicated and sufficient” (2 points), the global ideal score being 16 for non-comparative studies. Two investigators performed the MINORS assessment twice (AI and AB) at an interval of 10 days; then, the scores were discussed whenever a difference was present until a consensus was reached. Categorization of the MINORS scores was performed based on the previous literature [35] as follows: “Very low” (0–4 points); “Low” (5–8 points); “Good” (9–12); and “Excellent” (10,14–16).

### 2.6. Synthesis of Results

Summarized data were presented as total numbers, percentages, means and ranges. All analyses were performed using the Stata statistical software package (StataCorp, Stata Statistical Software: Release 14. College Station, TX, USA: StataCorp LLC) A critical analysis was provided for each outcome, but a formal statistical approach was performed only if data in primary studies were sufficient. The trend in terms of type of study, level of evidence, body segment and medical condition was analyzed in the timeframe between 2013 and 2023.

## 3. Results

**Figure 1:**
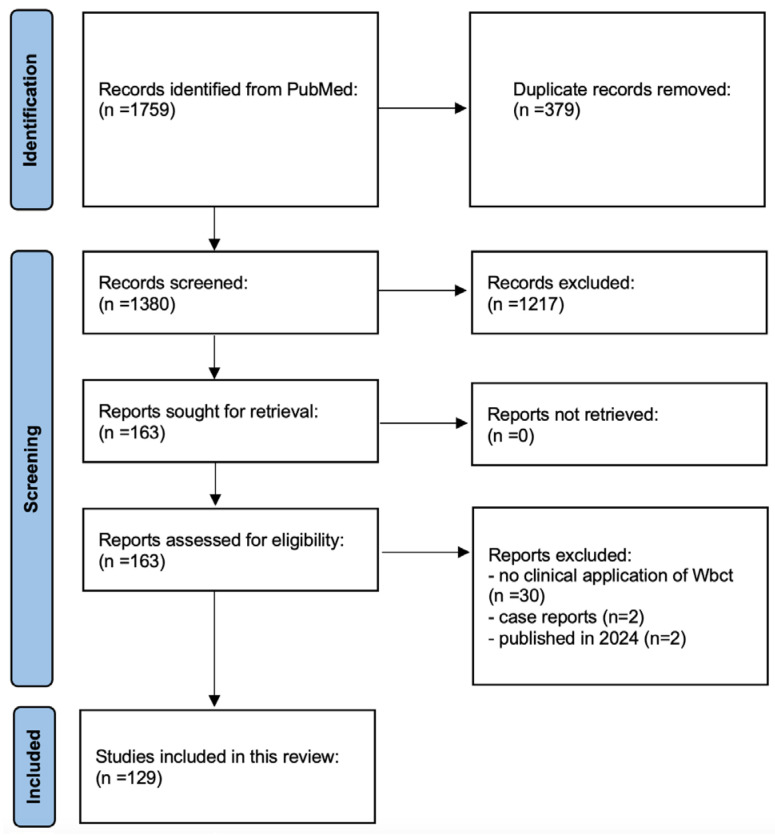
Flow chart.

**Figure 2:**
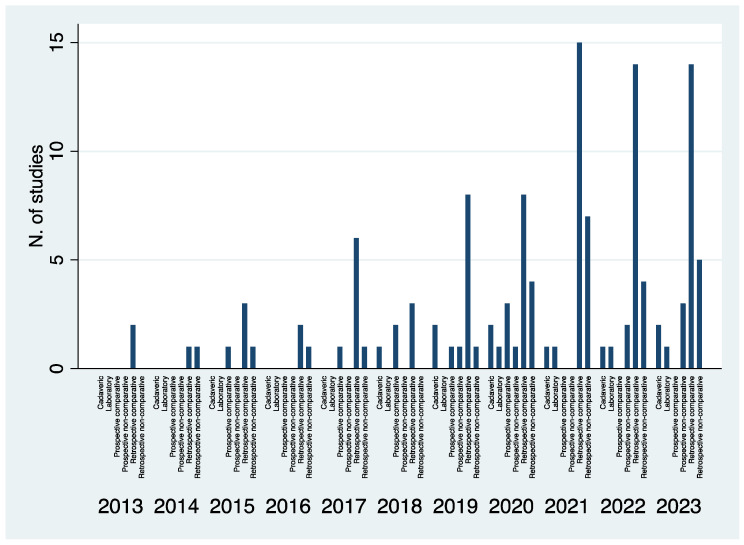
Histogram representing the number of studies published between 2013 and 2023 based on the study design.

**Figure 3:**
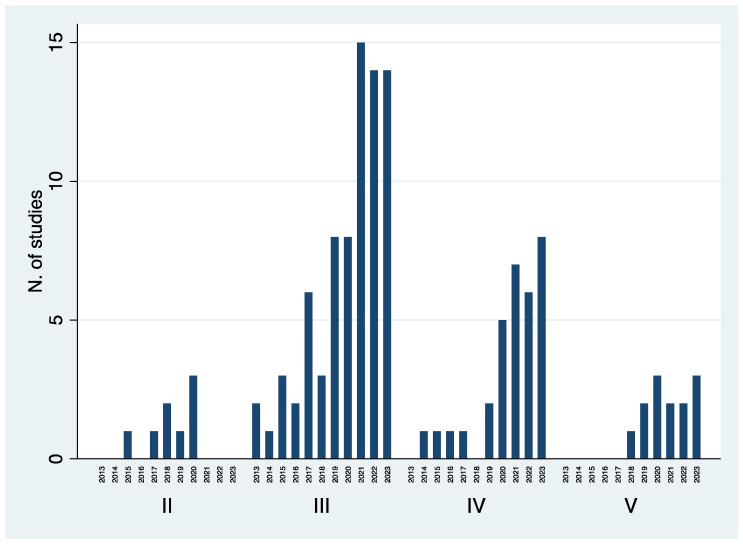
Histogram representing the number of studies published between 2013 and 2023 based on their level of evidence.

Out of 1759 studies, 129 followed the inclusion criteria and were selected (Figure 1) [36,37,38,39,40,41,42,43,44,45,46,47,48,49,50,51,52,53,54,55,56,57,58,59,60,61,62,63,64,65,66,67,68,69,70,71,72,73,74,75,76,77,78,79,80,81,82,83,84,85,86,87,88,88,89,90,91,92,93,94,95,96,97,98,99,100,101,102,103,104,105,106,107,108,109,110,111,112,113,114,115,116,117,118,119,120,121,122,123,124,125,126,127,128,129,130,131,132,133,134,135,136,137,138,139,140,141,142,143,144,145,146,147,148,149,150,151,152,153,154,155,156,157,158,159,160,161,162,163,164]. Nine studies dealt with acute conditions while 120 with chronic ones. The median year of publication was 2021 (IQR, 2019–2022; range 2013–2023). The median sample size was 25 cases (IQR, 15–48; range 3–1175) in the whole cohort, 26 cases (IQR, 16–55) in the Elective group and 20 (IQR, 13–26.5) in the Trauma group (*p* = 0.04). Retrospective comparative analyses (Level III) represented the most common study design during the whole timeframe analyzed (Figure 2). There was a progressive increase in the number of Level III, Level IV and Level V studies published over the years (Figure 3). The overall quality of studies was good with a mean MINORS score at 12 (range, 7 to 16) for studies on elective conditions and at 11 (range, 8 to 16) for studies on traumatic conditions. The devices used to perform WBCT scans have been reported in Table 1.

### 3.1. Elective Conditions

**Figure 4:**
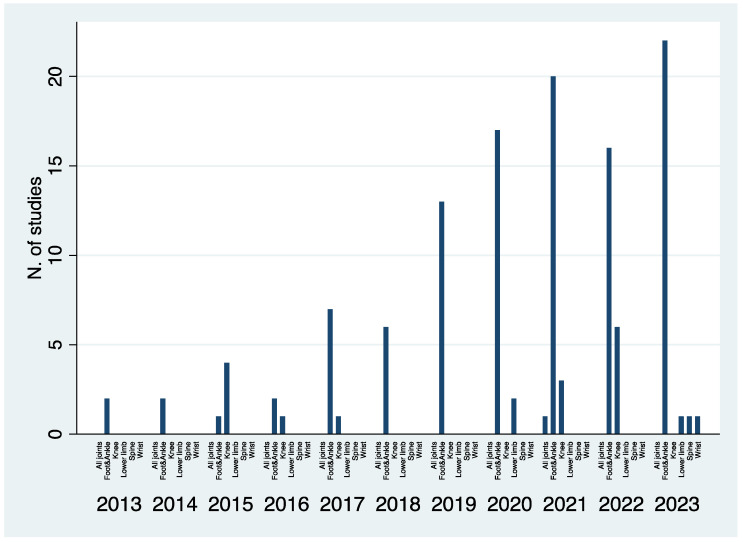
Histogram representing the number of studies published between 2013 and 2023 based on the anatomical area investigated in the same studies.

Out of 129, 105 (81%) manuscripts dealt with elective orthopedic conditions. Sixty-six papers (62%) were comparative by design, but only five (4.7%) were prospective (Table 2). The majority of the analyses (88 studies, 84%) were performed on foot and ankle conditions, while 13 studies (12%) looked at knee pathologies (Figure 4). A variety of diseases were investigated (as reported in Table 3) with particular interest in Progressive Collapsing Foot Deformity (22 studies; 25%) and Hallux Valgus (19 studies; 21%).

### 3.2. Traumatic Conditions

Out of 131, 24 (18%) manuscripts dealt with traumatic conditions. Seventeen papers (70%) were comparative by design, but only three (12.5%) were prospective (Table 2). The majority of analyses (20 studies; 83%) were performed on foot and ankle conditions, with two studies focusing on knee issues (8%) and one on lower limbs (4%) (Figure 4). The main ailments investigated are reported in Table 3, demonstrating particular interest in syndesmotic injuries (12 studies; 60%).

## 4. Discussion

In this review, including studies published between 2013 and 2023, we documented an increasing interest from the scientific community towards the clinical applications of weightbearing CT in the orthopedic field. Even if the majority of studies have been focusing on conditions related to the foot and the ankle, we also found a number of works investigating the value of this imaging modality for other joints (in particular, the knee), which probably depends on the technological advancements in the area with the availability of new machines. Of note, in the decade analyzed, we documented a ten-times increase in the number of studies between the first and the last two years (2 studies retrieved in 2013 and 2014 vs. 22 studies in 2022 and 26 studies in 2023) with a variety of conditions being investigated and with results suggesting that three-dimensional imaging during stance might enable clinicians to assess with greater accuracy musculoskeletal structures with a reduced amount of radiations due to the cone beam modality.

Considering that weightbearing imaging is often not requested in the trauma setting, it is not surprising that 81% of studies dealt with elective conditions. However, at least two considerations must be made around this point. First, it is now widely accepted that the diagnostic pathway for subtle ligamentous injuries of the lower limb must include imaging during stance in which the natural ‘stress’ applied on ligaments is reproduced through the application of gravity. This explains why almost one-fifth of studies in the foot and ankle field (among these, 14% and 5% regarded syndesmotic injuries and Lisfranc instability, respectively) focus on ligamentous lesions, which often risk going unseen on standard imaging [151]. Even if already before the advent of WBCT, there was agreement that syndesmotic lesions needed loading in order to be unmasked [165,166,167,168,169], it is also known that some subtle lesions at this level are considered easy to miss. This is where the the value of WBCT should be proven with more accurate measurements and greater diagnostic accuracy. In detail, regarding the syndesmosis, Borjali et al. have recently evaluated 48 patients with unstable syndesmotic lesions and compared them with 96 controls using deep learning models on WBCT images and demonstrated that a very high accuracy with a reduced time could be achieved [146]. A previous landmark meta-analysis published in 2022 by Raheman et al. on 11 studies using WBCT technology (including 559 ankles in 408 uninjured patients and 151 patients with syndesmotic instability) has confirmed that the so-called ‘syndesmotic area’ is amongst the most reliable parameters to diagnose syndesmotic instability, since it significantly increases under loading in injured ankles [170]. Similarly, bilateral standing radiographs have always been deemed necessary in order not to miss ligamentous Lisfranc injuries not associated with bony lesions [171,172,173,174], although, in this context, limits related to the bi-dimensionality of standard imaging are well known. On a different note, the limited number of studies dealing with urgent conditions possibly reflects the fact that the possibility to obtain tri-planar tomographic images under physiological load has prevailed in the mind of most users, while the use of cone beam non-weightbearing scans (potentially useful in all traumatic injuries of the lower limb, of the wrist or of the hand [52]) has not spread in clinical protocols yet. In a 2021 study by Jacques et al., cone beam CT was assessed in the context of an emergency radiology department, proving that the radiation dose was significantly reduced, and the turnover significantly accelerated as compared to the same months of the previous year (when only a standard multi-detector CT was available) [52].

In terms of level of evidence provided, there is no doubt that most studies were retrospective, with 80% among the elective studies and 64% among the trauma studies. As depicted in Figure 3, the number of Level III (retrospective comparative) and Level IV (retrospective non-comparative) studies has been steadily growing over the years. Conversely, we have noticed a low number of prospective comparative analyses (Level II) produced over time, while no randomized controlled trial has been published to date at all. In our opinion, this depends on the fact that most authors have aimed at investigating the role of WBCT as innovative imaging technology in the diagnosis and follow-up of orthopedic conditions, which is a necessary step to validate its use and allow its diffusion in clinical daily practice. Now that multiple analyses are being provided in different conditions, physicians have the basis to propose protocols for high-quality studies in which WBCT is a crucial tool of investigation and not the target of the study itself.

Looking at conditions analyzed in studies included in this review, it is clear that three-dimensional deformities like Progressive Collapsing Foot Deformity (PCFD) [115,126,128,129,137] and Hallux Valgus [62,70,71,72,75,76,78,79,163] have represented the most frequent pathologies evaluated using WBCT. These conditions involve abnormalities both in bone and soft tissue, making necessary a standing imaging in order to plan correctly any kind of surgical procedure. What is more, in this setting, rotation or torsion of a single segment may hugely vary between one patient and another, and only an appropriate assessment of the deformity may allow to plan surgical gestures in order to achieve the best outcome. The possibility to obtain such imaging with a reduced amount of radiation and a tomographic acquisition with subsequent three-dimensional reconstruction has been attractive for clinicians since the introduction of WBCT machines in the market. For what concerns Hallux Valgus, standing multiplanar imaging has certainly helped to better understand the degrees of rotation or torsion often associated with the well-known valgus deformities and theoretically associated with a certain risk of recurrence of the deformity. For what concerns studies related to the knee, it is not surprising that in all conditions investigated (i.e., osteoarthritis, patellar instability, knee instability and total knee replacement), the measurements taken in non-weightbearing conditions differed from those recorded using WBCT. It is worth mentioning that surgical planning before total knee replacement (or also total hip replacement) still relies on weightbearing radiographs (which do not allow us to consider properly any axial malalignment) or on non-weightbearing CT (which means unreal joint spaces due to the lack of gravity) [175,176,177,178,179,180], which might reduce the accuracy of planning itself and reduce the success rate and patient satisfaction.

It may be interesting to highlight that although, during the last years, WBCT machines able to scan the whole lower limb have been made available, we were unable to find studies dedicated to the hip, and we could select only some of them dealing with lower limb alignment. Most of them focused primarily on the relationship between hindfoot and lower limb alignment [108,111,112,181]. Before the introduction of WBCT (and also currently in centers where radiographs are the standard standing imaging), authors around the world have often focused on the complex relationship between the knee, the ankle and the heel, trying to establish which changes may occur in a joint when surgery is performed at a different level [33,182,183,184,185,186,187,188,189,190,191,192,193,194,195]. Amongst studies adopting WBCT, in 2023, Dufrenot et al. found an association between external tibial rotation and varus hindfoot in healthy people and hypothesized a sort of compensatory mechanism between knee and hindfoot alignments. However, they also highlighted that, in some other patients, this correlation was not present, therefore raising concerns about a potential failure of such mechanism in some cases [111]. In a similar study led by Burssens et al. in 2020, the authors detected differences between patients with and without tibiotalar osteoarthritis [181]. In detail, in patients with ankle osteoarthritis, a varus knee was associated with a valgus hindfoot, and a valgus knee was associated with a varus hindfoot. Conversely, patients without tibiotalar joint osteoarthritis presented with the same deviation at the level of the knee and hindfoot [181]. While active research is going on to explore the correlation between femoral torsion, knee and foot alignment, so far, results have not been unanimous, and a solid conclusion about hindfoot and suprasegmentary alignment cannot be drawn yet.

Based on what we found in the current literature, we think that some limitations of WBCT devices must be considered here. As an example, the initial cost of these machines has often been discussed as a potential limiting factor to the spreading of the technology in clinical centers. Not many cost-effectiveness studies have been published so far; however, in a population-based study published in 2021, two periods of time were compared in the emergency setting: a 7-month period during which only a standard multi-detector CT was available, and, one year later, an equivalent 7-month period during which a CBCT was also used [52]. The authors found a significantly reduced radiation dose and an accelerated turnover (23.6% faster) with CBCT in place. Based on this, and taking into account the need to reduce waiting lists in public hospitals, it is likely that initial economical effort to buy the machine would soon be compensated by the gain in terms of diagnostic workflow, as already discussed above. On a different note, whether the management of WBCT devices should be given to radiologists (as it could be given considering the nature of the device) or to orthopedic surgeons (who, according to the literature discussed above, are the main users of the device given the clinical advantages in diagnosis and, even more, surgical three-dimensional planning) has also been a matter of debate. While in an ideal setting, both specialties should collaborate and move in the same direction, the fact that this does not always happen and the lack of agreement felt by clinicians in daily practice are advocated as a further limitation to the acquisition and use of WBCT devices.

On a different note, some considerations should be made around the quality of images obtained using WBCT scans. The field of view of these machines generally includes billions of voxels, with a voxel size ranging between 0.25 and 0.4 mm, which allows to assess musculoskeletal structures satisfactorily. However, there are clinical scenarios, such as for the arthrodeses of small joints, which might pose a challenge for the clinician due to the size of spot welds between juxtaposed bony surfaces and the presence of metalwork in the area. It is the authors’ experience that, over the years, a great effort has been made by companies to further improve the quality of images, increasing the resolution of images and making available metal artifact reduction software in order to enable a correct evaluation of small anatomical areas as well.

Finally, with regard to the upper limb, our investigation found a single study by Buckwalter et al. in which the ulnar variance during handstands was assessed in ten gymnasts [161]. While we are aware that a greater number of studies have been published dealing with upper limb conditions and CBCT (such as scaphoid and wrist fractures [196,197,198,199,200,201,202,203]), we acknowledge that our research must have missed them due to the keyword ‘weightbearing’ used in the research process which has certainly limited the output. Even in this setting, multiple authors have underlined that a wider use of cone beam CT might allow for more time to reach a final diagnosis, avoiding double imaging (i.e., standard radiographs followed by CT in uncertain cases) and detecting occult bony lesions [202], such as scaphoid fractures [204,205,206,207,208,209,210]. Interestingly, concerning the spine, while we are aware of ongoing research activity to describe the value of weightbearing MRI [211,212,213,214,215,216], we found a single study by Feldle et al. in 2023 in which a gantry-free cone beam CT was used to scan the lumbar spine from eight cadaveric specimens to establish the most dose-effective combination of scan parameters [144]. Their study, in which an optimized protocol was established, will likely be used as a starting point for future analysis in this area. While industries are advancing fast to produce machines to scan the whole body in standing position, as of March 2024, we are already aware of cone beam tomographic devices presented on the market waiting for approval for clinical use worldwide.

## 5. Conclusions

In this review, we documented an increasing interest in clinical applications of weightbearing CT in the orthopedic field between 2013 and 2023. The majority of analyses has focused on three-dimensional complex conditions related to the foot and the ankle; however, we found several works investigating the value of WBCT for other joints (in particular the knee), also demonstrating a benefit from this technology for the assessment of suprasegmentary issues. Further work is warranted to confirm or disprove our findings.

## Figures and Tables

**Table 1:** List of devices used in studies in this review.

Company	Device	Body Parts Scanned	N. of Studies	%
Carestream	Onsight	Knee, ankle, foot, toes (single-leg stance, weightbearing); hand, wrist, forearm, elbow (non-weightbearing)	18	13.9
Curvebeam AI	HiRise	Whole lower limb (double-leg stance, weightbearing); hand, wrist, forearm, elbow (non-weightbearing)	1	0.7
Curvebeam AI	Pedcat	Ankle, foot, toes (double-leg stance, weightbearing)	82	63.6
Planmed Oy	Verity	Knee, ankle, foot, toes (single-leg stance, weightbearing); hand, wrist, forearm, elbow (non-weightbearing)	19	14.8
Not specified	-		9	7
			129	100

**Table 2:** Design of studies included in this review.

Design	Elective	%	Trauma	%
Prospective Comparative	5	4.8	3	12.5
Prospective Non-comparative	6	5.7	1	4.1
Retrospective Comparative	61	58.6	14	58.3
Retrospective Non-comparative	25	23.8	2	8.3
Cadaveric study	5	4.8	3	12.5
Laboratory study	3	2.8	1	4.1
Total	105		24	

**Table 3:** Anatomical structures and main conditions analyzed in studies included in this review.

Anatomical Area	Elective	%	Trauma	%
Foot and Ankle	88	83.8	20	82.6
Progressive Collapsing Foot Deformity	22	25		
Hallux Valgus	19	21.5		
Hindfoot alignment	13	14.7		
Ankle osteoarthritis	7	7.9		
Cavovarus foot	5	5.6		
Foot alignment	5	5.6		
Lower limb alignment	3	3.4		
Syndesmotic injury	3	3.4	12	60
Foot fractures	-		2	10
Hindfoot instability	2	2.2		
Lisfranc instability	2	2.2	3	15
Ankle instability	1	1.1	2	10
Hallux Rigidus	1	1.1		
Hindfoot osteoarthritis	1	1.1		
Muller-Weiss	1	1.1		
Pediatrics	1	1.1		
Pediatric deformities	1	1.1		
Pilon fracture	-		1	5
Windlass mechanism	1	1.1		
Knee	13	12.5	2	8.6
Osteoarthritis	7	6.7		
Patellar instability			2	8.3
Knee Alignment	3	2.8		
Knee instability	1	0.9		
Total Knee Replacement	2	1.9		
Lower Limb	2	1.9	1	4.1
Spine	1	0.9	-	
Wrist	1	0.9		
All Joints	-		1	4.1
Total	105		24	

## Data Availability

Data can be made available upon request to the corresponding author.
